# A multicentre, prospective, non-interventional study evaluating the safety of dapagliflozin in patients with type 2 diabetes in routine clinical practice in China (DONATE)

**DOI:** 10.1186/s12916-023-02906-7

**Published:** 2023-06-14

**Authors:** Lixin Guo, Jing Wang, Li Li, Lin Yuan, Sheng Chen, Hui Wang, Tonghuan Li, Lin Qi, Hong Yang

**Affiliations:** 1 Department of Endocrinology, Beijing Hospital, National Centre of Gerontology, Institute of Geriatric Medicine, Chinese Academy of Medical Sciences, No. 1, Dongdan Dahua Road, Dongcheng District, Beijing, 100730 People’s Republic of China; 2 Weifang Municipal Hospital, Weifang, People’s Republic of China; 3 The First Affiliated Hospital of Ningbo University, Ningbo, People’s Republic of China; 4 Zhuhai People’s Hospital, Zhuhai, People’s Republic of China; 5 The People’s Hospital of Liuyang, Liuyang, People’s Republic of China; 6 Yancheng Tinghu District People’s Hospital, Yancheng, People’s Republic of China; 7 The 81st Hospital of People’s Liberation Army, Nanjing, People’s Republic of China; 8 Present Address: The Second Hospital of Nanjing, Nanjing, People’s Republic of China; 9 Beijing Yanhua Hospital, Beijing, People’s Republic of China; 10 Rui’an People’s Hospital, Rui’an, People’s Republic of China

**Keywords:** Chinese, Dapagliflozin, Genital tract infection, Hypoglycaemia, Non-interventional study, Real world, Safety, Sodium-glucose cotransporter-2 inhibitors, Type 2 diabetes, Urinary tract infection

## Abstract

**Background:**

There are few large-scale studies evaluating the safety of the sodium-glucose cotransporter-2 inhibitor, dapagliflozin, in Chinese patients with type 2 diabetes. DONATE, a multicentre, single-arm, prospective, non-interventional study, is the first real-world study evaluating the safety of dapagliflozin in Chinese patients with type 2 diabetes in routine clinical practice.

**Methods:**

Between August 2017 and July 2020, patients with type 2 diabetes who had initiated dapagliflozin therapy and received ≥1 dose were prospectively recruited from 88 hospitals in China. Patients were subsequently followed up for 24 weeks; if patients discontinued dapagliflozin they were followed up for an additional 7 days after treatment discontinuation. The primary outcome was the proportion of patients with adverse events and serious adverse events, particularly key adverse events of special interest (AESI) including urinary tract infection, genital tract infection (typical symptoms with or without microbiological diagnosis) and hypoglycaemia (typical symptoms with or without blood glucose ≤3.9 mmol/L, or blood glucose ≤3.9 mmol/L without symptoms). Exploratory outcomes included the absolute change in metabolic parameters and the proportion of patients with other AESI including volume depletion, abnormal blood electrolytes, polyuria, renal impairment, diabetic ketoacidosis, hepatic impairment and haematuria.

**Results:**

A total of 3000 patients were enrolled, of whom 2990 (99.7%) were included in the safety analysis set. Mean (SD) age was 52.6 (12.0) years, and 65.8% of patients were male. Mean (SD) duration of type 2 diabetes at enrolment was 8.4 (7.1) years. Mean (SD) treatment duration of dapagliflozin was 209.1 (157.6) days. Adverse events were reported in 35.4% (*n* = 1059) of patients during the 24-week follow-up period. Overall, 9.0% (*n* = 268) were related to treatment and 6.2% (*n* = 186) were serious. Urinary tract infection, genital tract infection and hypoglycaemia were reported in 2.3% (*n* = 70), 1.3% (*n* = 39) and 1.1% (*n* = 32) of patients, respectively. The proportion of patients with other AESI was also low: polyuria (0.7%; *n* = 21), volume depletion (0.3%; *n* = 9), renal impairment (0.3%; *n* = 8), hepatic impairment (0.2%; *n* = 7), haematuria (0.2%; *n* = 6) and diabetic ketoacidosis (0.1%; *n* = 2).

**Conclusions:**

This study demonstrated that once-daily dapagliflozin was well tolerated in Chinese patients with type 2 diabetes and the overall safety profile of dapagliflozin in clinical practice in China was consistent with that reported in clinical trials.

**Trial registration:**

ClinicalTrials.gov, NCT03156985. Registered on 16 May, 2017.

**Supplementary Information:**

The online version contains supplementary material available at 10.1186/s12916-023-02906-7.

## Background

Type 2 diabetes mellitus (T2DM) is characterised by insulin resistance and a progressive loss of beta cell function [1], and it independently increases the risk of vascular and renal death by 2.3- and three-fold, respectively [2]. China has the largest number of adults with diabetes worldwide (140.9 million individuals in 2021), with an estimated overall prevalence of 12.4% in 2018 [3, 4]. Management of T2DM requires multifactorial behavioural and pharmacological treatment to manage blood-glucose levels, weight, and cardiovascular risk factors while ensuring cardiorenal protection [5]. Despite the availability of a wide variety of glucose-lowering medications, many patients with T2DM are inadequately treated and do not achieve glycaemic control (glycated haemoglobin [HbA_1c_] <7.0%), highlighting the need for more effective T2DM management [6, 7].

Sodium-glucose cotransporter-2 inhibitors (SGLT2i) are a novel class of glucose-lowering agents with a unique insulin-independent mechanism of action [8]. Dapagliflozin is a highly selective, orally active SGLT2i approved in 2017 by the Chinese National Medical Products Administration (NMPA) to improve glycaemic control in adults with T2DM. International and Chinese guidelines recommend SGLT2i for patients with T2DM with a compelling need to minimise hypoglycaemia and/or weight gain or to promote weight loss [9, 10]. Additionally, SGLT2i are recommended for patients at increased risk of cardiovascular events, with established atherosclerotic cardiovascular disease, with heart failure or with chronic kidney disease in light of evidence for the cardiovascular and renal benefits of SGLT2i in cardiovascular outcome trials [5, 9, 10].

Several adverse events (AEs) were associated with SGLT2i, albeit some (such as urinary and genital tract infections and volume depletion) are expected due to the specific class mechanism of action [9, 11, 12]. Nonetheless, safety data from clinical trials in international [13, 14] and Asian and Chinese [15, 16] populations have demonstrated that dapagliflozin treatment in patients with T2DM has a favourable and predictable safety profile. However, there is still a lack of large-scale studies evaluating the safety of dapagliflozin in Chinese patients with T2DM [15, 16]. In China, the NMPA requires that the safety of all newly approved drugs be assessed in ≥3000 patients within the first 5 years after approval. As such, the primary objective of the DONATE study was to evaluate the safety of dapagliflozin by assessment of AEs during a 24-week follow-up in Chinese patients with T2DM in clinical practice.

## Methods

### Study design and participants

DONATE was a multicentre, single-arm, prospective, non-interventional study (ClinicalTrials.gov Identifier: NCT03156985). Chinese patients were consecutively enrolled during routine clinical visits between 16 August 2017 and 30 July 2020 from 88 secondary and tertiary hospitals across eight regions in China. Eligible patients were required to have been diagnosed with T2DM according to the 2013 Chinese guidelines for diabetes treatment [17]: typical diabetes symptoms (e.g., polydipsia, polyuria, polyphagia, weight loss) with random plasma glucose ≥11.1 mmol/L and/or fasting plasma glucose ≥7.0 mmol/L and/or 2-h post-challenged plasma glucose ≥11.1 mmol/L; in patients without typical symptoms testing was repeated on a separate day. Patients were also required to have been prescribed dapagliflozin by their physician according to clinical practice, and to have received ≥1 dose of dapagliflozin (see Additional file 1: Table S1 for a full list of eligibility criteria) [17]. Dapagliflozin treatment was confirmed at the first study visit; patients already receiving dapagliflozin prior to enrolment were also eligible. The dosing of dapagliflozin and concomitant medications was at the discretion of the physicians according to current clinical practice [18]. After enrolment, patients were followed up for 24 weeks; if dapagliflozin was discontinued during the study period, patients were followed up for an additional 7 days after discontinuation. Treatment discontinuation was based on the patient’s own decision or the physician’s professional discretion. Patients who discontinued dapagliflozin treatment prior to study completion were followed up via telephone contact if a face-to-face visit was not feasible.

The study comprised three on-site visits: at enrolment (Day 0), at 12 weeks ± 7 days (Week 12) and at 24 weeks ± 7 days (Week 24) (Additional file 1: Table S2 and Additional file 1: Fig. S1). The last observation prior to the first dose of dapagliflozin treatment was used as the baseline measurement. If there was no value prior to the first dose of study treatment, then the baseline value was set to missing. Patient demographics, clinical characteristics and medical and diabetes history were recorded at enrolment (Additional file 1: Table S2). AEs and serious AEs (SAEs) were collected from the time of granting informed consent until 7 days after the last visit. Information on vital signs, laboratory tests and concomitant medication was collected, if available, at enrolment, Week 12 and Week 24 (Additional file 1: Table S2). Prior and concomitant antidiabetic medications were coded by the WHO Drug Dictionary [19].

The study was designed and conducted in accordance with the Declaration of Helsinki, Good Clinical Practice guidelines and local clinical practice regulations in China. Ethics approval was provided by the Institutional Review Board of Beijing Hospital (2017BJYYEC-054-02). Written informed consent was obtained from all patients prior to study screening. Patients were reimbursed for incurred travel expenses up to the value of 300 RMB (100 RMB per visit).

### Outcomes

The primary endpoint was the proportion of patients with AEs and SAEs, particularly key adverse events of special interest (AESI), including hypoglycaemia (typical symptoms with or without blood glucose ≤3.9 mmol/L, or blood glucose ≤3.9 mmol/L without symptoms), urinary tract infections (UTIs), and genital tract infections (GTIs; typical symptoms with or without microbiological diagnosis). AEs were coded using the Medical Dictionary for Regulatory Activities (MedDRA). The definitions of AEs, SAEs and adverse drug reactions (ADRs) are described in Additional file 1: Table S3. The definitions of individual AESIs are described in Additional file 1: Table S4 [20]. Exploratory endpoints included the proportion of patients who experienced other AESI (defined in Additional file 1: Table S4) as well as the absolute change in HbA_1c_, fasting plasma glucose (FPG), 2-h postprandial plasma glucose (2h-PPG), body weight, waist circumference, blood pressure and the proportion of patients achieving HbA_1c_ <7.0%.

### Statistical analysis

A target sample size of 3000 patients was determined based on the post-authorisation drug intensive monitoring programme criteria. Based on a previous study in Chinese patients, the proportion of patients with UTI, GTI and hypoglycaemia following treatment with dapagliflozin was 3.9–5.3%, 3.1–4.5% and 0.8%, respectively [21]. If the proportion of patients with any AE is assumed at 50.0%, 3000 evaluable patients will achieve a precision (or half-width) of 1.8%; if the proportion of patients with any AE is assumed at 0.8%, the precision achieved from 3000 evaluable patients is 0.3%. Accordingly, we estimated that 3000 patients would be sufficient to derive a 95% probability of observing at least one case if the proportion of patients with any AE is ≥0.1%.

The safety analysis set (SAS) was used as the primary analysis set for all safety analyses and included all patients who received ≥1 dose of dapagliflozin. The metabolic analysis set (MAS) included all patients who were treated with dapagliflozin continuously for ≥90 days and had baseline data with at least one post-baseline datapoint available.

For continuous variables, mean, median, standard deviation (SD) and range were calculated, while percentages were calculated for categorical variables. No imputation method for missing data was utilised. Change from baseline was calculated as the post-baseline assessment value minus the baseline assessment value; if either value was missing, the change from baseline was also missing. The Kaplan–Meier method was used to estimate the incidence of hypoglycaemia, UTI and GTI at Week 12 and Week 24. Estimated time to incidence was defined as the time from the first dose of dapagliflozin to the onset of a specific AE. Patients were censored on the day of their last dose of dapagliflozin or discontinuation of the study, whichever happened first. Univariate and multivariate Cox regression models were performed to further explore the association of patient characteristics with the incidence of hypoglycaemia, UTI and GTI. AEs were grouped according to AE severity and relationship to study treatment. Statistical analysis was performed using Statistical Analysis System version 9.4.

## Results

### Patient demographics and characteristics

**Fig. 1:**
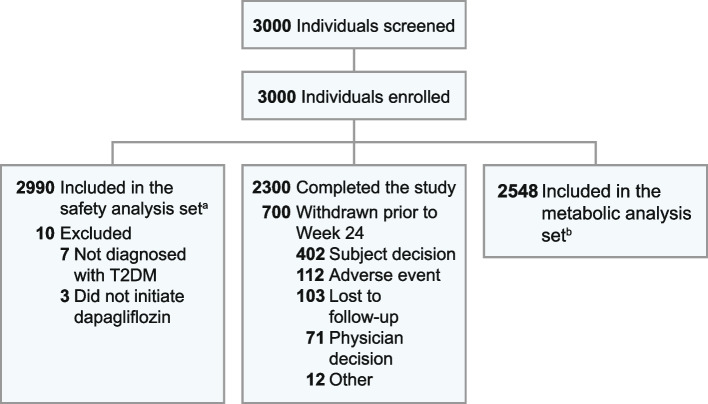
Patient disposition. ^a^All patients who received at least one dose of dapagliflozin. ^b^Patients who were treated with dapagliflozin consistently for at least 90 days. T2DM, type 2 diabetes mellitus

In total, 3000 patients were enrolled (Fig. 1). Of these, 2990 (99.7%) received ≥1 dose of dapagliflozin and were included in the SAS, while 2548 (84.9%) were included in the MAS. A total of 700 (23.3%) enrolled patients withdrew from the study; the main reasons for withdrawal were patients’ own decision (13.4%), AEs (3.7%) and loss to follow-up (3.4%).

Patient demographics and clinical characteristics at study enrolment (SAS) are shown in Table 1. Briefly, patients were aged (mean [SD]) 52.6 (12.0) years, with 8.35 (7.1) years of diabetes duration. Most patients were male (65.8%). Patients had a mean (SD) HbA_1c_ and FPG of 9.1 (2.0)% and 10.22 (4.0) mmol/L, respectively.

**Table 1:** Patient demographics and clinical characteristics at study enrolment (safety analysis set)

Characteristics	Dapagliflozin (*N* = 2990)
Age, years^a^	
No. (missing)	2990 (0)
Mean (SD)	52.6 (12.0)
Male	
No. (missing)	2990 (0)
No. (%)	1966 (65.8)
HbA_1c_^b^	
No. (missing)	1286 (1722)
%, mean (SD)	9.07 (2.0)
FPG, mmol/L	
No. (missing)	1314 (1676)
Mean (SD)	10.22 (4.0)
Body weight, Kg	
No. (missing)	408 (2582)
Mean (SD)	76.90 (13.9)
Height, cm	
No. (missing)	2879 (111)
Mean (SD)	167.42 (8.2)
BMI, Kg/m^2^	
No. (missing)	403 (2587)
Mean (SD)	27.27 (4.0)
Median (range)	26.80 (16.9–45.2)
BMI category, n (%)^c^	
≤18.5	2 (0.5)
>18.5 and <24	64 (15.9)
≥24 and <28	189 (46.9)
≥28	148 (36.7)
Waist circumference, cm	
No. (missing)	255 (2635)
Mean (SD)	96.21 (10.5)
SBP, mmHg	
No. (missing)	407 (2583)
Mean (SD)	131.6 (15.6)
DBP, mmHg	
No. (missing)	407 (2583)
Mean (SD)	81.0 (10.1)
Total cholesterol, mmol/L	
No. (missing)	1299 (1691)
Mean (SD)	4.87 (1.6)
HDL-C, mmol/L	
No. (missing)	1297 (1693)
Mean (SD)	1.15 (0.4)
LDL-C, mmol/L	
No. (missing)	1299 (1691)
Mean (SD)	2.79 (1.05)
Triglycerides, mmol/L	
No. (missing)	1301 (1689)
Mean (SD)	2.83 (3.2)
eGFR, ml/min/1.73 m^2^	
No. (missing)	1298 (1692)
Mean (SD)	126.3 (41.1)
<45	3 (0.2)
≥45 and <60	25 (1.9)
≥60 and <90	183 (14.1)
≥90	1087 (83.7)
Duration of T2DM, years^d^	
No. (missing)	2932 (58)
Mean (SD)	8.35 (7.1)
Presence of cardiac disorders, n (%)	
ASCVD	640 (21.4)
Heart failure	37 (1.2)
Concomitant antidiabetic medications, n (%)	
None	152 (5.1)
Monotherapy	2838 (94.9)
Dual therapy	2240 (74.9)
Triple therapy	1168 (39.1)
Presence of diabetic complications	
No. (missing)	2990 (0)
No. (%)	1764 (59.0)

*ASCVD* atherosclerotic cardiovascular disease, *DBP* diastolic blood pressure, *FPG* fasting plasma glucose, *c* cholesterol, *SBP* systolic blood pressure, *T2DM* type 2 diabetes mellitus

^a^Age was calculated as: (date of informed consent − date of birth + 1)/365.25. ^b^To convert to mmol/mol: (HbA_1c_ % – 2.15) × 10.929. ^c^BMI was categorized into underweight (BMI ≤18.5), normal weight (>18.5–<24.0), overweight (≥24.0–28.0), and obese (≥28.0). ^d^T2DM duration (years) was calculated as: (date of informed consent − date T2DM was first diagnosed + 1)/365.25

At study enrolment, patients included in the MAS had a mean (SD) HbA_1c_, FPG and 2 h-PPG of 9.0 (1.94)%, 10.15 (3.98) mmol/L and 15.75 (5.10) mmol/L, respectively. Patient body weight, body mass index (BMI) and waist circumference (mean [SD]) were 77.10 (13.89) Kg, 27.23 (4.00) Kg/m^2^ and 95.94 (10.32) cm, respectively. Mean (SD) systolic blood pressure (SBP) and diastolic blood pressure (DBP) for the enrolled patients were 131.60 (15.53) mmHg and 81.20 (10.21) mmHg, respectively.

### Real-world use of dapagliflozin and concomitant medications

Mean (SD) dapagliflozin treatment duration was 209.1 (157.6) days. Most patients (2989/2990) received the preferred once-daily dose of dapagliflozin. Details on exposure to dapagliflozin, including exposure among patients with/without dapagliflozin treatment prior to study enrolment, are summarised in Additional file 1: Table S5.

In the SAS, 94.9% of patients received concomitant antidiabetic therapy (Additional file 1: Table S6 [19]. See Additional file 1: Table S7 for the most frequently prescribed concomitant glucose-lowering medications.

### AEs

A total of 1059 (35.4%) patients in the SAS reported ≥1 AE during the 24-week follow-up period (Table 2). This was similar between patients with exposure to dapagliflozin before enrolment (35.5%) and patients without exposure before enrolment (35.1%) (Additional file 1: Table S8). Among the overall population, 186 (6.2%) experienced SAEs and 268 (9.0%) reported ADRs, as assessed by study investigators. The most common AEs (MedDRA preferred terms) were upper respiratory tract infection (3.6%), UTI (2.1%) and constipation (1.4%); the majority of AEs were mild in severity (Table 3). The most common SAEs were inadequate control of T2DM (0.7%), diabetic ketosis (0.3%) and pneumonia (0.3%) (Table 4). The most common ADRs were UTI (1.6%), hypoglycaemia (0.6%), weight decreased (0.6%) and diabetic ketosis (0.5%) (Table 4). Overall, 4.7% of patients discontinued dapagliflozin due to AEs; the most common AEs leading to treatment discontinuation were UTI (0.5%) and weight decreased (0.3%) (Table 4); all patients who discontinued due to weight decreased had a baseline BMI <28 kg/m^2^.

**Table 2:** Overall summary of adverse events and adverse events of special interest (safety analysis set)

Adverse events	Dapagliflozin (*N* = 2990)
Overall summary, No. (%)	
≥1 AE	1059 (35.4)
≥1 ADR^a^	268 (9.0)
≥1 SAE	186 (6.2)
AE leading to discontinuation	141 (4.7)
AE of special interest, No. (%)	
UTI	70 (2.3)
GTI	39 (1.3)
Hypoglycaemia	32 (1.1)
Volume depletion	9 (0.3)
Abnormal blood electrolytes	0
Polyuria	21 (0.7)
Renal impairment	8 (0.3)
Diabetic ketoacidosis	2 (0.1)
Hepatic impairment	7 (0.2)
Haematuria	6 (0.2)

*ADR* adverse drug reaction, *AE* adverse event, *GTI* genital tract infection, *SAE* serious adverse event, *UTI* urinary tract infection

^a^AEs with a causality assessment designated as ‘yes’ were considered to be ADRs

**Table 3:** Summary of adverse events by severity and relationship to study treatment (safety analysis set)

AEs occurring in >0.5% of patients, No. (%)	Mild	Moderate	Severe	Related to study drug	Total
Infections and infestations	228 (7.6)	40 (1.3)	9 (0.3)	2 (0.1)	277 (9.3)
Upper respiratory tract infection	100 (3.3)	8 (0.3)	1 (0.0)	0	109 (3.6)
Bronchitis	30 (1.0)	4 (0.1)	0	0	34 (1.1)
Conjunctivitis	24 (0.8)	5 (0.2)	0	0	29 (1.0)
Gastrointestinal disorders	204 (6.8)	34 (1.1)	9 (0.3)	41 (1.4)	247 (8.3)
Constipation	36 (1.2)	5 (0.2)	2 (0.1)	8 (0.3)	43 (1.4)
Diarrhoea	26 (0.9)	6 (0.2)	0	4 (0.1)	32 (1.1)
Metabolism disorders	183 (6.1)	22 (0.7)	11 (0.4)	56 (1.9)	216 (7.2)
Hyperlipidaemia	34 (1.1)	1 (0.0)	0	2 (0.1)	35 (1.2)
Hyperuricaemia	31 (1.0)	2 (0.1)	0	0	33 (1.1)
Hypoglycaemia	31 (1.0)	0	0	19 (0.6)	31 (1.0)
Renal and urinary disorders	126 (4.2)	15 (0.5)	2 (0.1)	74 (2.5)	143 (4.8)
Urinary tract infection	56 (1.9)	7 (0.2)	0	49 (1.6)	63 (2.1)
Musculoskeletal and connective tissue disorders	117 (3.9)	22 (0.7)	1 (0.0)	8 (0.3)	140 (4.7)
Nervous system disorders	115 (3.8)	18 (0.6)	4 (0.1)	7 (0.2)	137 (4.6)
Dizziness	35 (1.2)	1 (0.0)	0	2 (0.1)	36 (1.2)
Reproductive system and breast disorders	85 (2.8)	12 (0.4)	1 (0.0)	45 (1.5)	98 (3.3)
Investigations	75 (2.5)	7 (0.2)	1 (0.0)	35 (1.2)	83 (2.8)
General administration site conditions disorders	70 (2.3)	10 (0.3)	2 (0.1)	21 (0.7)	82 (2.7)
Skin and subcutaneous tissue disorders	70 (2.3)	9 (0.3)	0	10 (0.3)	79 (2.6)
Eye disorders	56 (1.9)	9 (0.3)	4 (0.1)	1 (0.0)	69 (2.3)
Cardiac disorders	42 (1.4)	13 (0.5)	5 (0.2)	3 (0.1)	60 (2.0)

Patients were counted only once within an SOC and PT; if a patient reported multiple AEs within an SOC or PT, the most serious AE (for severity assessment) and the most frequent AE related to study drug (for assessment of relationship with study drug) were included. AEs are coded using MedDRA, version 23.1

*AE* adverse event, *MedDRA* Medical Dictionary for Regulatory Activities, *PT* preferred term, *SOC* system organ class

**Table 4:** Summary of serious adverse events, adverse drug reactions, and AEs leading to discontinuation (safety analysis set)

Occurring in ≥0.2% of patients, No. (%)	Dapagliflozin (*N* = 2990)
≥ 1 SAE	186 (6.2)
Metabolism and nutrition disorders	34 (1.1)
Inadequate control of T2DM^a^	20 (0.7)
Diabetic ketosis	8 (0.3)
Hyperglycaemia	5 (0.2)
Infections and infestations	26 (0.9)
Pneumonia	8 (0.3)
Nervous system disorders	23 (0.8)
Cerebral infarction	6 (0.2)
Cardiac disorders	21 (0.7)
Coronary artery disease	7 (0.2)
Gastrointestinal disorders	15 (0.5)
Large intestine polyp	5 (0.2)
Eye disorders	14 (0.5)
Musculoskeletal and connective tissue disorders	12 (0.4)
Intervertebral disc protrusion	5 (0.2)
Injury, poisoning and procedural complications	9 (0.3)
Neoplasms	8 (0.3)
Renal and urinary disorders	7 (0.2)
Vascular disorders	7 (0.2)
Hypertension	5 (0.2)
General disorders and administration site conditions	6 (0.2)
Hepatobiliary disorders	5 (0.2)
Reproductive system and breast disorders	5 (0.2)
≥1 ADR	268 (9.0)
Renal and urinary disorders	74 (2.5)
Urinary tract infection	49 (1.6)
Metabolism and nutrition disorders	56 (1.9)
Hypoglycaemia	19 (0.6)
Diabetic ketosis	15 (0.5)
Reproductive system and breast disorders	45 (1.5)
Vulvovaginal pruritus	13 (0.4)
Vaginal infection	10 (0.3)
Gastrointestinal disorders	41 (1.4)
Dry mouth	11 (0.4)
Investigations	35 (1.2)
Weight decreased	17 (0.6)
General disorders and administration site conditions	21 (0.7)
Asthenia	9 (0.3)
Thirst	5 (0.2)
Skin and subcutaneous tissue disorders	10 (0.3)
Musculoskeletal and connective tissue disorders	8 (0.3)
Nervous system disorders	7 (0.2)
Vascular disorders	5 (0.2)
AE leading to discontinuation	
Renal and urinary disorders	29 (1.0)
Urinary tract infection	14 (0.5)
Gastrointestinal disorders	25 (0.8)
Dry mouth	5 (0.2)
Reproductive system and breast disorders	25 (0.8)
Vaginal infection	7 (0.2)
Vulvovaginal pruritus	6 (0.2)
Metabolism and nutrition disorders	19 (0.6)
Diabetic ketosis	7 (0.2)
Inadequate control of T2DM	6 (0.2)
Investigations	16 (0.5)
Weight decreased	9 (0.3)
General disorders and administration site conditions	10 (0.3)
Asthenia	5 (0.2)
Skin and subcutaneous tissue disorders	10 (0.3)
Pruritis	5 (0.2)
Musculoskeletal and connective tissue disorders	7 (0.2)

AEs with a causality assessment designated as ‘yes’ were considered to be ADRs. Patients were counted only once within an SOC and PT; if a patient reported multiple AEs within an SOC or PT, the most serious AE (for severity assessment) and the most frequent AE related to study drug (for assessment of relationship with study drug) were included. AEs are coded using MedDRA, version 23.1

*ADR* adverse drug reaction, *AE* adverse event, *MedDRA* Medical Dictionary for Regulatory Activities, *PT* preferred term, *SAE* serious adverse event, *SOC* system organ class, *T2DM* type 2 diabetes mellitus

^a^Inadequate control of T2DM was evaluated against personalised treatment targets for individual patients

### AESIs

UTI, GTI and hypoglycaemia were reported in 70 (2.3%), 39 (1.3%) and 32 (1.1%) patients (Table 2), with 1.6%, 0.1% and 0.6% patients reporting events related to treatment, respectively (Table 3). UTI, GTI and hypoglycaemia were reported in 31 (1.6%), 12 (0.6%) and 23 (1.2%) male patients, and 39 (3.8%), 27 (2.6%) and 9 (0.9%) female patients. The proportion of patients experiencing other AESIs was relatively low (0.1%–0.7%), and no abnormal electrolyte events were reported (Table 2).

The estimated incidence of UTI, GTI and hypoglycaemia for the first 12 weeks was 0.9% (95% confidence interval [CI]: 0.645–1.349), 0.6% (95% CI: 0.376–0.946) and 0.6% (95% CI: 0.349–0.902), respectively; and for 24 weeks, the incidence was 1.7% (95% CI: 1.301–2.270), 1.0% (95% CI: 0.672–1.407) and 0.8% (95% CI: 0.491–1.131).

In the multivariate analysis, sex showed a predictive trend for incidence of UTI (hazard ratio [HR] 2.244, 95% CI: 1.047–4.810) and GTI (HR 6.723, 95% CI: 2.135–21.167) (Table 5). Presence of T2DM complications showed a predictive trend for GTI (HR 0.153 95% CI: 0.048–0.481), as did diabetes duration (>5 years and ≤10 years vs ≤5 years: HR 6.107, 95% CI: 1.212–30.785; >20 years vs ≤5 years: HR 20.580, 95% CI: 3.448–122.828) (Table 5).

**Table 5:** Analysis of risk factors for hypoglycaemia, urinary tract infection and genital tract infection (safety analysis set)

	**Univariate analysis**			**Multivariate analysis**^**a**^	
	**No.**	**HR (95% CI)**	***p* value**	**HR (95% CI)**	***p* value**
**Hypoglycaemia**					
Age (>65 years vs 18–65 years)	2985	0.682 (0.206–2.260)	0.531	0.526 (0.107–2.573)	0.428
Sex (female vs male)^b^	2986	0.913 (0.413–2.017)	0.821	1.254 (0.445–3.533)	0.669
BMI (vs ≥18.5 and <24)	2852				
≥24 and <28		0.920 (0.350–2.421)	0.866	0.591 (0.162–2.149)	0.424
≥28		0.715 (0.248–2.064	0.536	0.847 (0.230–3.129)	0.804
Diabetes duration (per increase of 1 year)	2928	1.064 (1.020–1.109)	0.004	–	–
Diabetes duration (vs ≤ 5 years)					
>5 and ≤10 years		9.317 (2.065–42.035)	0.004	3.530 (0.670–18.593)	0.137
>10 and ≤20 years		4.893 (1.039–23.054)	0.045	2.418 (0.437–13.376)	0.312
>20 years		14.995 (3.108–72.349)	<0.001	5.932 (0.962–36.557)	0.055
Presence of T2DM complications (yes vs no)^c,d^	2986	1.451 (0.656–3.208)	0.358	1.212 (0.369–3.981)	0.751
eGFR (1 ml/min/1.73 m^2^) (vs ≥90)	1763				
≥60 and <90		1.743 (0.568–5.350)	0.331	1.697 (0.528–5.455)	0.375
<60		0.000 (0.000–NE)	0.991	0.000 (0.000–NE)	0.990
Concomitant anti-diabetes medications (vs ≤1)	2986				
2		1.426 (0.429–4.735)	0.562	3.038 (0.337–27.406)	0.322
≥3		2.647 (0.885–7.919)	0.082	2.318 (0.219–24.577)	0.485
Insulin use (yes vs no)^c^	2986	1.882 (0.890–3.979	0.098	1.760 (0.594–5.218)	0.308
Sulfonylurea use (yes vs no)	2986	1.841 (0.850–3.989)	0.122	1.303 (0.365–4.658)	0.684
Metformin use (yes vs no)	2986	1.792 (0.681–4.714)	0.237	1.292 (0.320–5.222)	0.719
Presence of ASCVD (yes vs no)	2986	0.967 (0.391–2.388)	0.942	1.821 (0.637–5.206)	0.263
**Urinary tract infection**					
Age (>65 years vs 18–65 years)	2986	1.023 (0.522–2.004)	0.947	1.007 (0.322–3.151)	0.991
Sex (female vs male)^b^	2987	2.274 (1.406–3.678)	<0.001	2.244 (1.047–4.810)	0.038
BMI (vs ≥18.5 and <24)	2853				
≥24 and <28		1.782 (0.823–3.860)	0.143	1.483 (0.483–4.551)	0.491
≥28		1.642 (0.737–3.658)	0.225	1.468 (0.455–4.741)	0.521
Diabetes duration (per increase of 1 year)	2929	1.015 (0.983–1.048)	0.367	–	–
Diabetes duration (vs ≤5 years)					
>5 and ≤10 years		1.053 (0.552–2.007)	0.876	1.556 (0.580–4.178)	0.380
>10 and ≤20 years		0.985 (0.543–1.785)	0.959	1.303 (0.487–3.485)	0.598
>20 years		1.240 (0.533–2.882)	0.618	0.818 (0.159–4.210)	0.810
Presence of T2DM complications (yes vs no)^c,d^	2987	1.073 (0.656–1.755)	0.780	1.025 (0.439–2.392)	0.954
HbA_1c_ % (per increase of 1%)	1609	1.035 (0.882–1.214)	0.675	1.010 (0.781–1.306)	0.940
FPG (per increase of 1 mmol/L)	1823	1.023 (0.945–1.108)	0.568	1.054 (0.923–1.204)	0.438
eGFR (1 ml/min/1.73 m^2^) (vs ≥90)	1763				
≥60 and <90		1.479 (0.680–3.219)	0.324	1.387 (0.507–3.796)	0.524
< 60		0.000 (0.000–NE)	0.986	0.000 (0.000–NE)	0.989
**Genital tract infection**					
Age (> 65 years vs 18–65 years)	2984	0.780 (0.275–2.215)	0.641	0.757 (0.153–3.738)	0.733
Sex (female vs male)^b^	2985	4.006 (1.951–8.229)	<0.001	6.723 (2.135–21.167)	0.001
BMI (vs ≥18.5 and <24)	2851				
≥24 and <28		1.475 (0.405–5.371)	0.556	2.543 (0.344–18.790)	0.360
≥28		3.678 (1.091–12.395)	0.036	6.701 (0.948–47.380)	0.057
Diabetes duration (per increase of 1 year)	2927	1.039 (0.996–1.084)	0.076	–	–
Diabetes duration (vs ≤5 years)					
>5 and ≤10 years		1.698 (0.674–4.278)	0.262	6.107 (1.212–30.785)	0.028
>10 and ≤20 years		1.158 (0.457–2.935)	0.756	4.184 (0.743–23.560)	0.105
>20 years		2.824 (1.003–7.954)	0.049	20.580 (3.448–122.828)	< 0.001
Presence of T2DM complications (yes vs no)^c,d^	2985	0.321 (0.156–0.659)	0.002	0.153 (0.048–0.481)	0.001
HbA_1c_ % (per increase of 1%)	1609	0.878 (0.692–1.115)	0.286	1.101 (0.771–1.572)	0.597
FPG (per increase of 1 mmol/L)	1823	0.971 (0.863–1.093)	0.626	0.897 (0.724–1.112)	0.322
eGFR (1 ml/min/1.73 m^2^) (vs ≥90)	1764				
≥60 and <90		0.203 (0.026–1.621)	0.133	0.298 (0.037–2.432)	0.258
<60		0.000 (0.000–NE)	0.990	0.000 (0.000–NE)	0.992

CIs of HRs are Wald CIs. *p* values are nominal and based on Wald chi-square tests

*BMI* body mass index, *eGFR* estimated glomerular filtration rate, *FPG* fasting plasma glucose, *HbA*_*1c*_ glycated haemoglobin, *NE* not evaluable, *T2DM* type 2 diabetes mellitus

^a^*n* = 1681, *n* = 1158 and *n* = 1158 analysed in multivariate analyses for hypoglycemia, UTI and GTI, respectively. ^b^Reference group = male. ^c^Reference group = no. ^d^*n* = 1764 with T2DM complications (*n* = 23 with hypoglycemia, *n* = 44 with UTI, and *n* = 13 with GTI), and *n* = 1226 without T2DM complications (*n* = 9 with hypoglycemia, *n* = 26 with UTI, and *n* = 26 with GTI)

### Change in metabolic factors (MAS)

At Week 12, mean (SD) change from baseline in HbA_1c_, FPG and 2h-PPG was –1.522 (1.794)%, –2.022 (3.498) mmol/L and –1.967 (7.882) mmol/L, respectively. At Week 24, mean (SD) change from baseline was –1.318 (1.871)%, –1.826 (3.470) mmol/L and –5.466 (5.473) mmol/L, respectively (Additional file 1: Figs. S2-4). Mean (SD) body weight, BMI and waist circumference were 74.90 (13.34) Kg, 26.51 (3.73) Kg/m^2^ and 93.34 (10.45) cm at Week 12, and 74.64 (12.88) Kg, 26.37 (3.57) Kg/m^2^ and 92.87 (10.17) cm at Week 24, respectively. Mean (SD) SBP and DBP were 127.50 (13.72) mmHg and 78.90 (9.27) mmHg at Week 12, and 127.40 (13.18) mmHg and 78.50 (8.72) mmHg at Week 24, respectively. Mean (SD) changes from baseline for all metabolic factors and vital signs are shown in Additional file 1: Figs. S2–9. The proportions of patients achieving HbA_1c_ <7.0% and HbA_1c_ <7.0% without hypoglycaemia are shown in Additional file 1: Fig. S10.

## Discussion

This is the largest non-interventional study undertaken in Chinese patients with T2DM evaluating the safety of dapagliflozin in clinical practice, as well as the largest study investigating the safety of any SGLT2i in China to date. Results from this observational study show that dapagliflozin treatment was associated with a low frequency of AEs, including AESIs, demonstrating a favourable safety profile and tolerability in Chinese patients with T2DM in a real-world setting. Overall, no new safety findings were reported and the safety profile of dapagliflozin was consistent with that established in dapagliflozin clinical trials [22–25].

Overall, baseline patient demographics and clinical characteristics, including metabolic factors and vital signs, were similar to those reported in two Phase 3 studies evaluating the safety of dapagliflozin in Asian (predominantly Chinese) populations [15, 16]. However, there were notable differences, including the longer duration of T2DM, and increased HbA_1c_, body weight, waist circumference, SBP and DBP in our study. These differences suggest that in a real-world setting, Chinese patients with T2DM receive dapagliflozin at a more advanced stage of the disease and have poorer glycaemic control and metabolic markers than patients with T2DM included in clinical trials.

Most patients received a once-daily dose of dapagliflozin and had a mean treatment duration of 209.1 days, indicating good real-world treatment compliance. The majority of patients (94.9%) received concomitant antidiabetic therapy during the study, most frequently metformin (68.3%). The dapagliflozin and metformin combination is widely used in clinical practice together with lifestyle management in patients with T2DM, indicating that the study population was representative of the real world in terms of clinical management [22].

In the present study, the proportion of patients reporting AEs of any grade (35.4%) was lower than that found in clinical trials in international (60.0–61.7%), Asian (53.6–58.7%) and Chinese (52.4–61.7%) populations [14–16, 26, 27], confirming that dapagliflozin is well tolerated in Chinese patients with T2DM in clinical practice. We hypothesise that this difference could be due to physicians using a high degree of caution when selecting appropriate patients, as dapagliflozin had only recently launched in China at the time of study initiation and was the only SGLT2i with approval.

Several prespecified AESIs were carefully monitored during the 24-week follow-up period. These AESIs are potentially associated with treatment with dapagliflozin and other SGLT2i [11], and some of these appear to be related to dapagliflozin’s mechanism of action [26, 28, 29]. Overall, the proportion of patients experiencing ≥1 AESI in this study was low (6.4%), including UTI, GTI and hypoglycaemia (2.3%, 1.3% and 1.1%, respectively). In a study of patients treated with dapagliflozin in Korea, GTI and hypoglycaemia were reported in 3.4% and 13.0% of patients, respectively [30]. In pooled analyses of international dapagliflozin clinical trials, the proportions of patients reporting hypoglycaemia, UTI and GTI were 10.2–13.7%, 4.3–4.7% and 4.8–5.5%, respectively [14, 26], although these AESIs appear to be less frequent in Asian (1.6–1.9%, 3.5–4.7% and 1.8–2.6%, respectively) [27] and Chinese (0.0–1.4%, 3.6–6.6% and 0.9–‍2.0%, respectively) [15, 16] populations. The low proportion of patients reporting UTI and GTI in DONATE may have been influenced by the predominantly male population (65.8% male), although the proportion of male patients is consistent with that reported in dapagliflozin and empagliflozin pooled safety analyses (57.5%–65.4%) [14, 31]. It is possible, however, that a higher degree of caution was used by clinicians when considering dapagliflozin treatment in females due to the known risk of GTI and UTI and the recent approval of dapagliflozin in China at the time of study initiation. A meta-analysis of six randomised, placebo-controlled trials (*N* = 2033; up to 24 weeks of follow-up) showed that dapagliflozin treatment was associated with an increased relative risk of 1.74 (95% CI: 1.21–2.49; *p* = 0.003) for UTI and 3.52 (95% CI: 2.06–6.03; *p* <1 × 10^–5^) for GTI [32]. Multivariate analyses in the present study showed that the incidence of UTI and GTI in females was increased by approximately 2.2- and 6.7-fold, respectively, compared with males. Sex has previously shown a strong association with UTI and GTI in patients with T2DM receiving dapagliflozin [28, 29, 33], although this has also been shown in patients with and without T2DM irrespective of dapagliflozin treatment [34]. Results of the DONATE study suggest that the incidence of UTI, GTI and hypoglycaemia is low in clinical practice in China (estimated incidence: 0.9%, 0.6% and 0.6% at Week 12, and 1.7%, 1.0% and 0.8% at Week 24, respectively). Nonetheless, we found that UTI was the most frequent AE leading to treatment discontinuation.

We additionally found that longer duration of diabetes was associated with increased incidence of GTI, but was not associated with incidence of UTI or hypoglycemia. However, an association between disease duration and hypoglycaemia has been previously shown in patients with T2DM [35], which could be due to a decline in islet function over time. Treatment with SGLT2i improves glycaemic control independent of insulin secretion [8], which may account for differences between these studies. Another study has reported no association between diabetes duration and GTI [33]; this study also reported no association between HbA_1c_ and GTI, but there is evidence that poor glycaemic control may increase incidence of GTI [36, 37]. It is interesting that in DONATE, longer duration of diabetes but not glycemic control (measured by HbA_1c_) was associated with incidence of GTI. Although, while HbA_1c_ reflects average blood glucose levels over 3 months, this measurement may not fully reflect the impact of longer-term glucose toxicity. In the setting of uncontrolled glucose levels, patients with T2DM have impaired immune system function and thus are at increased risk of infections [38]. Therefore, good control of blood glucose levels and regular check-ups may help to reduce the risk of GTI. Further research is required to confirm the risk factors for GTI in patients with diabetes, particularly for patients treated with SGLT2i given the increased risk conferred by treatment [14, 39, 40]. In the present study, the proportion of patients reporting other AESIs was low, including the frequency of diabetic ketoacidosis (0.1%). A UK study reported the frequency of diabetic ketoacidosis in patients treated with dapagliflozin in clinical practice to be 1.9% [41]. Of note, the proportion of patients with renal impairment was lower than in the pooled safety analysis by Jabbour and colleagues (0.3% vs 0.8%) [14].

This study, which provides a comprehensive real-world analysis of dapagliflozin treatment in routine clinical practice, identified ‘weight decreased’ as an adverse drug reaction in 17 patients. While weight loss is an expected and desirable effect of SGLT2i [42], it may be perceived differently from patient to patient and could potentially be deemed an adverse effect by some, especially those who are elderly or not overweight/obese. Clinicians should be aware of the potential unintended consequences of excessive or rapid weight loss, including malnutrition and anxiety. However, weight loss is a significant metabolic effect of dapagliflozin and even modest reductions in weight can lead to improvements in glycemia and other cardiovascular risk factors [43]. In DONATE, where the majority of patients were classified as overweight or obese, dapagliflozin treatment resulted in an overall decrease in mean weight over the study period.

Other metabolic parameters of interest such as HbA_1c_, FPG and 2h-PPG improved throughout the 24-week follow-up, although all results using the metabolic analysis set should be interpreted with caution due to substantial missing data. The mean (SD) absolute change from baseline in HbA_1c_ at Week 24 was consistent with that observed in clinical practice in the UK (–1.06 [1.49]%) [41]. An improvement was also observed in the proportion of patients achieving HbA_1c_ <7.0% throughout the present study. This is consistent with two randomised clinical trials of Chinese patients treated with dapagliflozin 10 mg, in which HbA_1c_ <7.0% was achieved in 33.0−49.8% of patients at the 24-week follow-up [15, 16]. In a real-world analysis of US databases evaluating glycaemic control in patients with T2DM, 25.1% of patients treated with dapagliflozin 10 mg achieved HbA_1c_ <7.0% at 6 months [44]. Current integrated glycaemic control targets for T2DM in China include HbA_1c_ <7.0% [17, 45]. Results of the DONATE study suggest that dapagliflozin treatment resulted in an increase in the proportion of patients with T2DM meeting this criteria, demonstrating its valuable role in the integrated management of patients with T2DM in a real-world setting [17].

Strengths of the study include the large sample size and wide variety of contemporary clinical practice settings across diverse regions of China. However, the results should be viewed in the context of the following limitations. Firstly, data on AEs prior to study enrolment were not collected retrospectively; therefore, early AEs may have been omitted from the analyses due to the time of data collection. Secondly, there was a limited number of patients with available post-baseline data for all parameters. Patients who provided data may have been those with a high level of diabetes management compliance; thus, data from the present study should be interpreted with caution. The lack of available post-baseline records for the change in metabolic parameters and vital signs suggests that patients with T2DM may not be routinely followed up in clinical practice in China. A recent focus group study evaluating the encounters between Chinese general practitioners (GPs) and patients with T2DM identified key challenges that GPs face, from short consultation time to inadequate patient information resources and healthcare support [46]. These issues may explain the lack of periodic follow-up in the DONATE study. Thirdly, the effects of concomitant glucose-lowering medications cannot be easily separated from the effects of dapagliflozin. Fourthly, while there are many interesting observations in DONATE, the data we collected does not allow us to investigate them all. For instance, we did not collect data on the reason(s) for initiation of dapagliflozin and so we are unable to evaluate adherence to the local clinical guidelines [18]. In addition, we did not collect detailed information on diabetes complications, which hinders interpretation of the association of diabetes complications with risk of GTI. We encourage further research in these areas. Finally, and as with observational studies in general, confounding could have influenced the results.

## Conclusions

In conclusion, dapagliflozin use under varied clinical practice settings in China was associated with a favourable safety profile and a low incidence of AEs, especially those of special interest (GTI, UTI and hypoglycaemia). Moreover, improvements were observed in glycaemic control and other metabolic parameters.

## Supplementary Information

**Additional file 1: Table S1.** Eligibility criteria. **Table S2.** Schedule of visits and data collection. **Table S3.** Outcome definitions. **Table S4.** Definition of adverse events of special interest. **Table S5.** Summary of dapagliflozin exposure. **Table S6.** Concomitant medications. **Table S7.** Most common concomitant antidiabetic medications. **Table S8.** Overall summary of adverse events and adverse events of special interest by timing of exposure to dapagliflozin. **Fig. S1.** Study design. **Fig. S2.** Mean change from baseline in HbA_1c_ during the 24-week study follow-up. **Fig. S3.** Mean change from baseline in FPG during the 24-week study follow-up. **Fig. S4.** Mean change from baseline in 2h-PPG during the 24-week study follow-up. **Fig. S5.** Mean change from baseline in body weight during the 24-week study follow-up. **Fig. S6.** Mean change from baseline in BMI during the 24-week study follow-up. **Fig. S7.** Mean change from baseline in waist circumference during the 24-week study follow-up. **Fig. S8.** Mean change from baseline in SBP during the 24-week study follow-up. **Fig. S9.** Mean change from baseline in DBP during the 24-week study follow-up. **Fig. S10.** Proportion of patients achieving HbA_1c_ <7.0% throughout the 24-week study follow-up.

## Data Availability

The datasets used and/or analysed during the current study are available from the corresponding author on reasonable request.

## Abbreviations

ADR: Adverse drug reaction
AE: Adverse event
AESI: Adverse event of special interest
BMI: Body mass index
CI: Confidence interval
DBP: Diastolic blood pressure
FPG: Fasting plasma glucose
GP: General practitioner
GTI: Genital tract infection
HR: Hazard ratio
MAS: Metabolic analysis set
MedDRA: Medical Dictionary for Regulatory Activities
NMPA: National Medical Products Administration
2h-PPG: 2-Hour postprandial plasma glucose
SAE: Serious adverse event
SAS: Safety analysis set
SBP: Systolic blood pressure
SD: Standard deviation
SGLT2i: Sodium-glucose cotransporter-2 inhibitors
T2DM: Type 2 diabetes mellitus
UTI: Urinary tract infection
