# Supplementary material for: A multicentre, prospective, non-interventional study evaluating the safety of dapagliflozin in patients with type 2 diabetes in routine clinical practice in China (DONATE)
Source: BMC Med. 2023 Jun 14;21:212. doi: 10.1186/s12916-023-02906-7 (PMC10268430; doi:10.1186/s12916-023-02906-7)
Supplement: Supplementary file 1 — Additional file 1: Table S1. Eligibility criteria. Table S2. Schedule of visits and data collection. Table S3. Outcome definitions. Table S4. Definition of adverse events of special interest. Table S5. Summary of dapagliflozin exposure. Table S6. Concomitant medications. Table S7. Most common concomitant antidiabetic medications. Table S8. Overall summary of adverse events and adverse events of special interest by timing of exposure to dapagliflozin. Fig. S1. Study design. Fig. S2. Mean change from baseline in HbA1c during the 24-week study follow-up. Fig. S3. Mean change from baseline in FPG during the 24-week study follow-up. Fig. S4. Mean change from baseline in 2h-PPG during the 24-week study follow-up. Fig. S5. Mean change from baseline in body weight during the 24-week study follow-up. Fig. S6. Mean change from baseline in BMI during the 24-week study follow-up. Fig. S7. Mean change from baseline in waist circumference during the 24-week study follow-up. Fig. S8. Mean change from baseline in SBP during the 24-week study follow-up. Fig. S9. Mean change from baseline in DBP during the 24-week study follow-up. Fig. S10. Proportion of patients achieving HbA1c <7.0% throughout the 24-week study follow-up. [file 12916_2023_2906_MOESM1_ESM.docx]

**Additional File 1**

**A multicentre, prospective, non-interventional study evaluating the safety of dapagliflozin in patients with type 2 diabetes in routine clinical practice in China (DONATE)**

Lixin Guo, MD;^1^ Jing Wang, MMed;^2^ Li Li, MMed;^3^ Lin Yuan, MMed;^4^ Sheng Chen, MMed;^5^ Hui Wang, MMed;^6^ Tonghuan Li, MD;^7,*^ Lin Qi, MMed;^8^ Hong Yang, MMed^9^

^1^ Beijing Hospital, Beijing, People's Republic of China

^2^ Weifang Municipal Official Hospital, Shandong, People's Republic of China

^3^ Ningbo First Hospital, Zhejiang, People's Republic of China

^4^ Zhuhai People’s Hospital, Guangdong, People's Republic of China

^5^ The People’s Hospital of Liuyang, Hunan, People's Republic of China

^6^ Yancheng Tinghu People’s Hospital, Jiangsu, People's Republic of China

^7^ The 81^st^ Hospital of People’s Liberation Army, Jiangsu, People's Republic of China

^8^ Beijing Yanhua Hospital, Beijing, People's Republic of China

^9^ Zhejiang Rui’an People’s Hospital, Zhejiang, People's Republic of China

^*^Tonghuan Li was affiliated to 81st Hospital of People’s Liberation Army at the time of the trial and is currently affiliated to The Second Hospital of Nanjing.

**Corresponding author**

Lixin Guo

Address: Department of Endocrinology, Beijing Hospital, National Centre of Gerontology, Institute of Geriatric Medicine, Chinese Academy of Medical Sciences, No. 1, Dongdan Dahua Road, Dongcheng District, Beijing 100730, People's Republic of China.

E-mail: [glx1218@163.com](mailto:glx1218@163.com)

Contents

[Supplementary Tables 3](#_Toc134802888)

[Table S1. Eligibility criteria 3](#_Toc134802889)

[Table S2. Schedule of visits and data collection 4](#_Toc134802890)

[Table S3. Outcome definitions 5](#_Toc134802891)

[Table S4. Definition of adverse events of special interest 6](#_Toc134802892)

[Table S5. Summary of dapagliflozin exposure (safety analysis set) 8](#_Toc134802893)

[Table S6. Concomitant medications (safety analysis set) 9](#_Toc134802894)

[Table S7. Most common concomitant antidiabetic medications (safety analysis set) 10](#_Toc134802895)

[Table S8. Overall summary of adverse events and adverse events of special interest by timing of exposure to dapagliflozin (safety analysis set) 12](#_Toc134802896)

[Supplementary figures 13](#_Toc134802897)

[Figure S1. Study design. 13](#_Toc134802898)

[Figure S2. Mean change from baseline in HbA1c during the 24-week study follow-up (metabolic analysis set). 14](#_Toc134802899)

[Figure S3. Mean change from baseline in FPG (mmol/L) during the 24-week study follow-up (metabolic analysis set). 15](#_Toc134802900)

[Figure S4. Mean change from baseline in 2h-PPG (mmol/L) during the 24-week study follow-up (metabolic analysis set). 16](#_Toc134802901)

[Figure S5. Mean change from baseline in body weight (Kg) during the 24-week study follow-up (metabolic analysis set). 17](#_Toc134802902)

[Figure S6. Mean change from baseline in BMI (Kg/m^2^) during the 24-week study follow-up (metabolic analysis set). 18](#_Toc134802903)

[Figure S7. Mean change from baseline in waist circumference (cm) during the 24-week study follow-up (metabolic analysis set). 19](#_Toc134802904)

[Figure S8. Mean change from baseline in SBP (mmHg) during the 24-week study follow-up (metabolic analysis set). 20](#_Toc134802905)

[Figure S9. Mean change from baseline in DBP (mmHg) during the 24-week study follow-up (metabolic analysis set). 21](#_Toc134802906)

[Figure S10. Proportion of patients achieving HbA1c <7.0% throughout the 24-week study follow-up (metabolic analysis set). 22](#_Toc134802907)

# Supplementary Tables

## Table S1. Eligibility criteria

| **Inclusion criteria** | **Exclusion criteria** |
| --- | --- |
| - Provision of patients’ informed consent prior to study enrolment. - Chinese female or male patient diagnosed with T2D, according to the 2013 Chinese guidelines for diabetes treatment (17) - Received ≥1 dose of dapagliflozin tablet 5 mg or 10 mg daily, prescribed by the physician according to standard clinical practice.^a^ | - Participation in any other clinical trial during the last 3 months prior to study enrolment. - Inability to fulfil the study-specified procedures. - Previous enrolment in the present study. |

^a^The prescription of dapagliflozin was independent of the decision to be included in the current study. FPG, fasting plasma glucose; T2D, type 2 diabetes.

## Table S2. Schedule of visits and data collection

|  | **Day 0^a^** | **12 weeks ±7 days** | **24 weeks ±7 days** |
| --- | --- | --- | --- |
| Informed consent | x |  |  |
| Inclusion and exclusion criteria | x |  |  |
| Demographics and clinical characteristics (age, sex, race, height, diabetes history, disease history) | x |  | x |
| Laboratory tests (if available)^b^ | x | x | x |
| Vital signs (weight, waist circumference, body mass index, blood pressure) | x | x | x |
| Concomitant medications | x | x | x |
| Adverse events | x | x | x |
| Reason for discontinuation |  | x | x |

^a^Baseline measurements were collected during the first visit. The last observation prior to the first dose of dapagliflozin treatment was used as the baseline measurement. If there was no value prior to the first dose of study treatment, then the baseline value was set to missing.
^b^Including biomarkers of glucose control and renal and liver function

## Table S3. Outcome definitions

| **Outcome** | **Definition** |
| --- | --- |
| Adverse event (AE) | - The development of any untoward medical occurrence or the deterioration of a pre-existing medical condition following initiation of study treatment, which does not necessarily have a causal relationship with study treatment. - The term AE includes both serious and non-serious AEs. |
| Serious adverse event (SAE) | - An AE that fulfils one or more of the following criteria:   - results in death   - is immediately life-threatening (life-threatening in this context refers to a reaction in which the patient was at risk of death at the time of the reaction; it does not refer to a reaction that hypothetically might have caused death if more severe)   - requires in-patient hospitalisation or prolongation of existing hospitalisation   - results in persistent or significant disability or incapacity   - is a congenital abnormality or birth defect   - is an important medical event that may jeopardise the patient or may require medical intervention to prevent one of the outcomes listed above. - Medical and scientific judgement should be exercised in deciding whether other situations should be considered an SAE. |
| Adverse drug reaction (ADR) | - A response to study treatment that is noxious and unintended. A response in this context means that a causal relationship between the medicinal product and an AE is at least a reasonable possibility. - AEs that have a causality assessment designated as yes will be considered ADRs. |

## Table S4. Definition of adverse events of special interest

| **Adverse event of interest** | **Definition** | | **MedDRA preferred term (events captured)** | |
| --- | --- | --- | --- | --- |
| **UTI** | - Diagnosis of UTI is at the discretion of physicians or investigators according to their own clinical practice - If urine cultures are available, data should be recorded in CRF | | Urinary tract infection, cystitis, *Escherichia* urinary tract infection, genitourinary tract infection, pyelonephritis, trigonitis, urethritis, kidney infection and prostatitis | |
| **GTI** | - Diagnosis of GTI is at the discretion of physicians or investigators according to their own clinical practice | | Vulvovaginal mycotic infection, vaginal infection, balanitis, genital infection fungal, vulvovaginal candidiasis, vulvovaginitis, candida balanitis, genital candidiasis, genital infection, genital infection male, penile infection, vulvitis, vaginitis bacterial and vulval abscess | |
| **Hypoglycaemia** | - Suspected of hypoglycaemia is defined as (1) typical symptoms such as trembling, palpitations, sweating, anxiety, hunger and (2) resolution of these symptoms and signs by carbohydrate ingestion - Clinical diagnosis of hypoglycaemia is defined as blood glucose ≤3.9 mmol/L with or without typical symptoms listed above | | Hypoglycaemia | |
| **Volume depletion** | - Diagnosis of volume depletion is at the discretion of physicians or investigators according to their own clinical practice | | Dehydration, hypovolaemia and hypotension | |
| **Abnormal blood electrolytes** | - Based on the laboratory tested value of blood electrolytes, such as potassium, sodium, chlorine, calcium, magnesium and phosphorus | Blood electrolytes abnormal, hypercalcaemia, hypocalcaemia, hyperphosphataemia, hypophosphataemia, hypernatraemia, hyponatraemia, hyperkalaemia and hypokalaemia | |  |
| **Polyuria** | - Diagnosis of polyuria is at the discretion of physicians or investigators according to their own clinical practice | Pollakiuria, polyuria and urine output increased | |  |
| **Renal impairment** | - Based on blood urea nitrogen, creatinine, uric acid and urinary protein test results - Mild (eGFR 60−90 ml/min/1.73 m^2^), moderate (eGFR 30−59 ml/min/1.73 m^2^) and severe (eGFR <30 ml/min/1.73 m^2^), according to KDIGO 2012 guidelines (20) | Blood creatinine abnormal/increased, creatinine renal clearance decreased, glomerular filtration rate abnormal/decreased, renal failure, acute kidney injury, chronic kidney disease, blood urea nitrogen/creatinine ratio increased, postrenal/prerenal failure and renal impairment | |  |
| **DKA** | - DKA was diagnosed at the discretion of the physician according to their own clinical practice; cases were reviewed and confirmed based on medical records and blood glucose, ketone, acid and electrolyte test results - Suspected events of DKA are defined as blood ketone <3.0 mmoL and/or ketone on urinalysis being negative | Diabetic ketoacidosis, ketoacidosis, ketosis, blood ketone body, blood ketone body increased/present, ketonuria, urine ketone body and urine ketone body present | |  |
| **Hepatic impairment** | - PHL and HL cases should be reported as SAE:   - PHL: AST or ALT ≥3x ULN and TBL ≥2x ULN at any point during the study, irrespective of an increase in ALP   - HL: AST or ALT ≥3x ULN and TBL ≥2x ULN where no reason other than IMP can be found to explain the combination of increases | Liver disorder, drug-induced liver injury, liver injury, hepatic failure, alanine aminotransferase increased, aspartate aminotransferase increased, hepatic lesion and hepatic enzyme increased | |  |
| **Haematuria** | - Confirmed by microscopy - If no immediate or benign cause is identified, such as menstruation, kidney stone and urinary tract infection where haematuria is subsequently resolved after successful treatment, patients should undergo further evaluation. The evaluation may include but is not limited to tests such as urine cytology, NMP-22 or abdominal CT scans. All confirmed events of bladder cancer should lead to the discontinuation of investigational product | Red blood cells urine positive, haematuria, blood urine and blood urine present | |  |

ALP, alkaline phosphatase; ALT, alanine aminotransferase; AST, aspartate aminotransferase; CRF, case report form; CT, computed tomography; DKA, diabetic ketoacidosis; GTI, genital tract infection; HL, Hy’s Law; IMP, investigational medicinal product; KDIGO, Kidney Disease Improving Global Outcomes; MedDRA, Medical Dictionary for Regulatory Activities; NMP-22, nuclear matrix protein 22; PHL, potential Hy’s Law; SAE, serious adverse event; TBL, total bilirubin; ULN, upper limit of normal; UTI, urinary tract infection.

##

## Table S5. Summary of dapagliflozin exposure (safety analysis set)

|  | **Patients with exposure to dapagliflozin before enrolment** | **Patients without exposure to dapagliflozin before enrolment** | **Dapagliflozin (Total)** |
| --- | --- | --- | --- |
| Exposure (days)^a^ |  |  |  |
| N (missing) | 2648 (0) | 342 (0) | 2990 (0) |
| Mean (SD) | 219.2 (163.1) | 130.8 (64.4) | 209.1 (157.6) |
| Median (range) | 185.0 (1–1774) | 166.5 (1, 241) | 182.0 (1–1774) |
| Q1, Q3 | 167.5, 233.0 | 89.0, 174.0 | 163, 223 |
| Exposure before enrolment^b^ |  |  |  |
| N (missing) | 2648 (0) | – | – |
| Mean (SD) | 73.4 (153.8) | – | – |
| Median (range) | 15.0 (2, 1597) | – | – |
| Q1, Q3 | 6.0, 64.0 | – | – |
| Dose frequency, n (%) |  |  |  |
| QD^c^ | 2647 (100) | 342 (100) | 2,989 (100) |
| QOD^d^ | 2 (0.1) | 0 | 2 (0.1) |
| BID | 3 (0.1) | 0 | 3 (0.1) |
| Reasons for dose adjustment, n (%) |  |  |  |
| Patient decision | 465 (17.6) | 73 (21.3) | 538 (18.0) |
| AE | 160 (6.0) | 30 (8.8) | 190 (6.4) |
| Glucose control | 168 (6.3) | 14 (4.1) | 182 (6.1) |
| Liver function | 3 (0.1) | 0 | 3 (0.1) |
| Other | 78 (2.9) | 5 (1.5) | 83 (2.8) |

^a^Exposure (days) = date of last dose − date of first dose + 1. ^b^Exposure (days) = date of informed consent – date of first dose + 1. ^c^Including every morning. ^d^Including every other night. AE, adverse event; BID, twice daily; QD, once daily; QOD, every other day.

## Table S6. Concomitant medications (safety analysis set)

| **Mono or combination therapy^a^** | **Dapagliflozin (N=2990)** |
| --- | --- |
| Antidiabetic medication class, n (%) | 2837 (94.9) |
| Biguanides | 2034 (68.0) |
| Sulphonylureas | 687 (23.0) |
| AGIs | 1009 (33.7) |
| DPP-4 inhibitors | 664 (22.2) |
| GLP-1 analogues | 326 (10.9) |
| Glitinides | 181 (6.1) |
| Thiazolidinediones | 221 (7.4) |
| SGLT2 inhibitors | 8 (0.3) |
| Metformin dual therapy | 180 (6.0) |
| ARIs | 1 (0.0) |
| Insulin analogues |  |
| Long acting | 864 (28.9) |
| Rapid acting | 358 (12.0) |
| Intermediate/long acting + rapid acting | 357 (11.9) |
| Human insulin |  |
| Rapid acting | 27 (0.9) |
| Intermediate acting | 11 (0.4) |
| Antihypertensive agents, n (%) | 1400 (46.8) |
| Other medication, n (%) | 2431 (81.3) |
| Traditional medicine | 743 (24.8) |
| Lipid-modifying agents | 1739 (58.2) |
| Antithrombotic agents | 1136 (38.0) |
| Cardiac therapy | 370 (12.4) |
| Systemic antibacterial agents | 274 (9.2) |

^a^Patients may have more than one medication record per drug class and preferred term. Patients were counted once if he/she reported one or more medication records within one medication class/group. Prior medications are defined as medications that started and ended prior to the first dose of dapagliflozin. Concomitant medications are defined as medications that ended after the first dose of dapagliflozin. Prior and concomitant antidiabetic medications were coded by the WHO Drug Dictionary (19). AGI, alpha-glucosidase inhibitor; ARI, aldose reductase inhibitor; DPP-4, dipeptidyl peptidase-4; GLP-1, glucagon-like peptide-1; SGLT2, sodium-glucose cotransporter-2.

## Table S7. Most common concomitant antidiabetic medications (safety analysis set)

| **Mono- or combination therapy** | **Dapagliflozin (N=2990)** |
| --- | --- |
| Metformin, n (%) | 2041 (68.3) |
| Dose (mg), n | 2037 |
| Mean (SD) | 714.3 (319) |
| Median (range) | 500 (100–3000) |
| Dose frequency, n (%) |  |
| QD^a^ | 180 (8.8) |
| BID | 929 (45.5) |
| TID | 786 (38.5) |
| QID | 75 (3.7) |
| Other | 71 (3.5) |
| Duration of treatment (days), n | 2041 |
| Mean (SD) | 1132.3 (1839.7) |
| Median (range) | 217 (1–10,735) |
| Acarbose, n (%) | 945 (31.6) |
| Dose (mg), n | 945 |
| Mean (SD) | 177.8 (3251.0) |
| Median (range) | 50 (1–100,000) |
| Dose frequency, n (%) |  |
| QD^a^ | 65 (6.9) |
| BID | 38 (4.0) |
| TID | 819 (86.7) |
| Other | 23 (2.4) |
| Duration of treatment (days), n | 945 |
| Mean (SD) | 842.1 (1479.5) |
| Median (range) | 192 (1–10,161) |
| Insulin glargine, n (%) | 651 (21.8) |
| Dose (U), n | 636 |
| Mean (SD) | 19.0 (7.7) |
| Median (range) | 18 (3–60) |
| Dose frequency, n (%) |  |
| QD^a^ | 456 (70.0) |
| BID | 3 (0.5) |
| TID | 2 (0.3) |
| Other | 190 (29.2) |
| Duration of treatment (days), n | 651 |
| Mean (SD) | 562.7 (1006.1) |
| Median (range) | 183 (1–7664) |

^a^Including every morning. Patients may have more than one medication record per drug class and preferred term. Patients were counted once for the largest dose he/she reported in one or more medication records. Duration of treatment = end date − start date + 1. Concomitant medications are defined as medications that ended after the first dose of dapagliflozin. BID, twice daily; TID, three times daily; QD, once daily; QID, four times daily.

## Table S8. Overall summary of adverse events and adverse events of special interest by timing of exposure to dapagliflozin (safety analysis set)

| **Adverse events, No. (%)** | **Patients with exposure to dapagliflozin before enrolment**  **(n=2648)** | **Patients without exposure to dapagliflozin before enrolment**  **(n=342)** |
| --- | --- | --- |
| Overall summary | | |
| ≥1 AE | 939 (35.5) | 120 (35.1) |
| ≥1 ADR^a^ | 234 (8.8) | 34 (9.9) |
| ≥1 SAE | 166 (6.3) | 20 (5.8) |
| AE leading to discontinuation | 112 (4.2) | 29 (8.5) |
| AE of special interest | | |
| UTIs | 56 (2.1) | 14 (4.1) |
| GTIs | 37 (1.4) | 2 (0.6) |
| Hypoglycaemia | 28 (1.1) | 4 (1.2) |
| Volume depletion | 6 (0.2) | 3 (0.9) |
| Abnormal blood electrolytes | 0 | 0 |
| Polyuria | 18 (0.7) | 3 (0.9) |
| Renal impairment | 7 (0.3) | 1 (0.3) |
| Diabetic ketoacidosis | 2 (0.1) | 0 |
| Hepatic impairment | 7 (0.3) | 0 |
| Haematuria | 3 (0.1) | 3 (0.9) |

ADR, adverse drug reaction; AE, adverse event; GTI, genital tract infection; SAE, serious adverse event; UTI, urinary tract infection. ^a^AEs with a causality assessment designated as ‘yes’ were considered to be ADRs.

# **Supplementary** **figures**

**
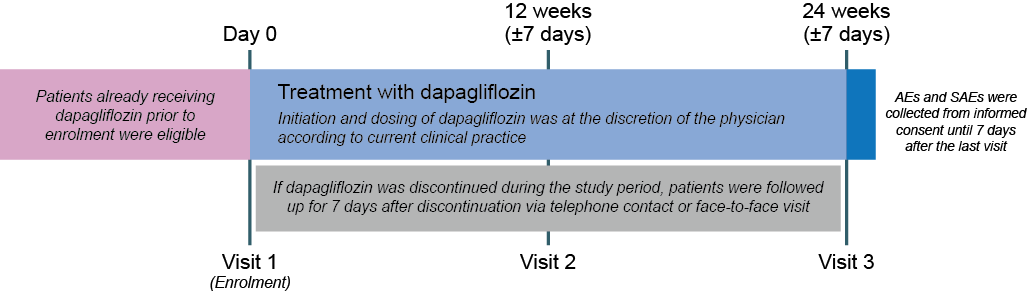
**

## Figure S1. Study design.

AE, adverse event; SAE, serious adverse event


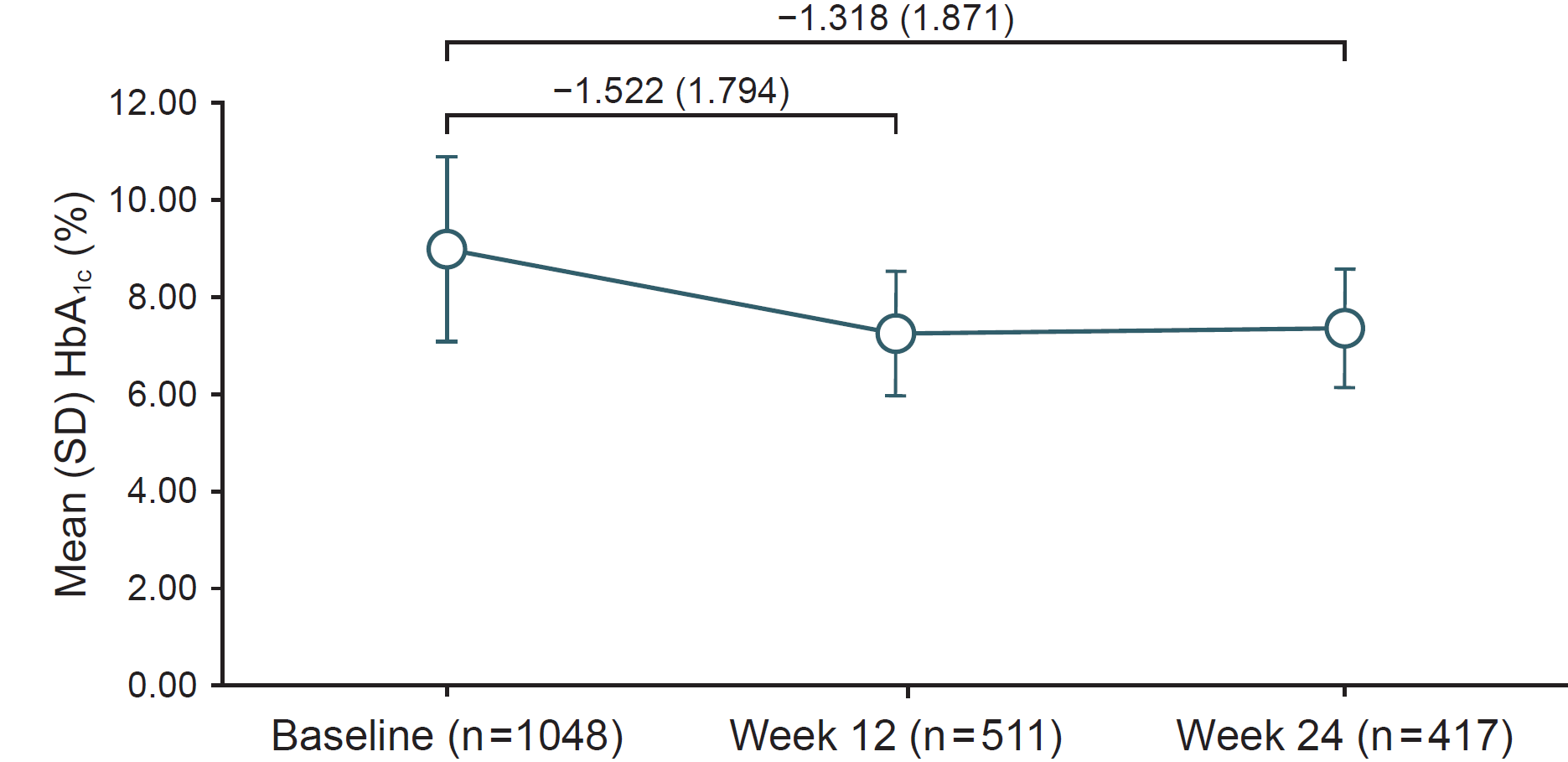


## Figure S2. Mean change from baseline in HbA1c during the 24-week study follow-up (metabolic analysis set).

Error bars represent SD.


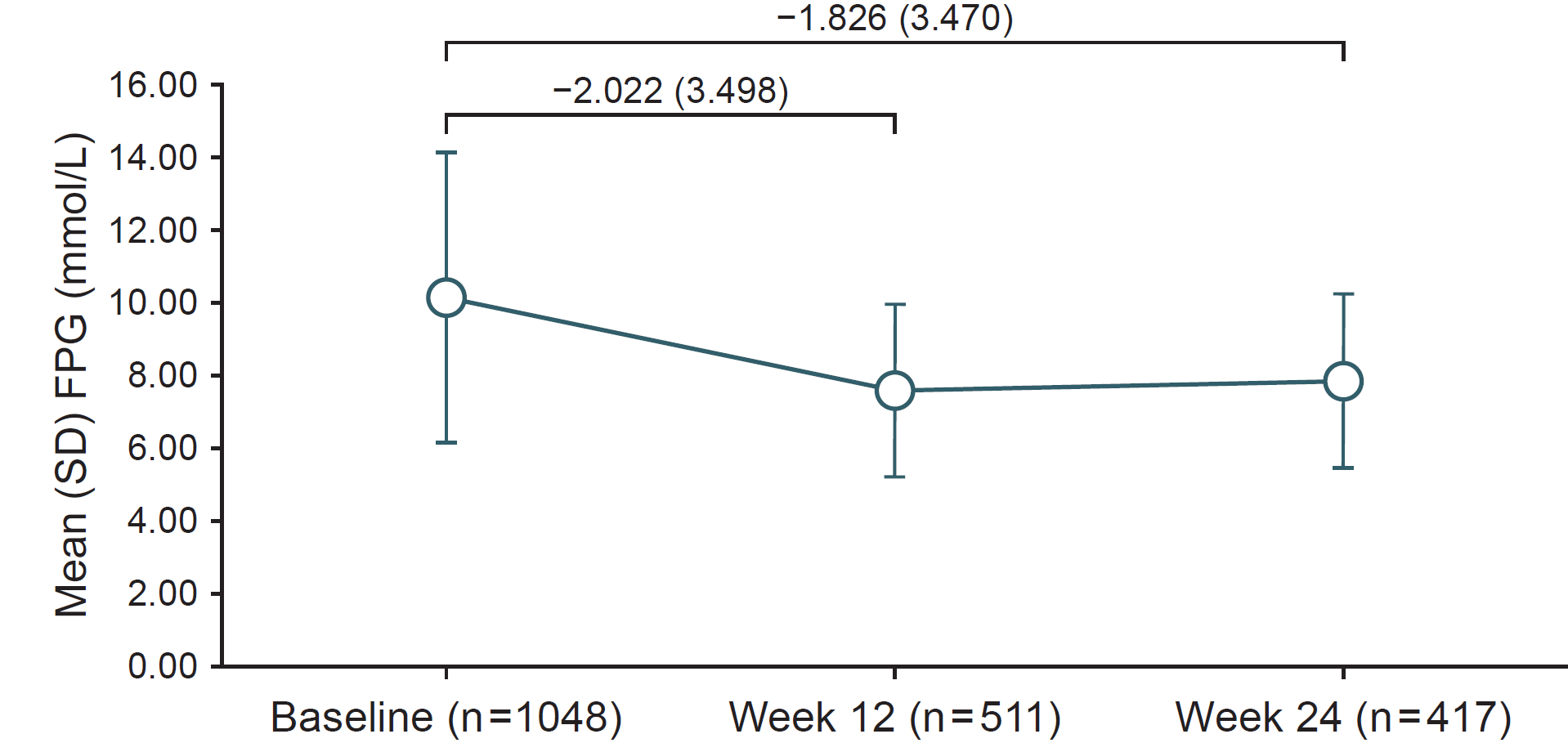


## Figure S3. Mean change from baseline in FPG (mmol/L) during the 24-week study follow-up (metabolic analysis set).

Error bars represent SD. FPG, fasting plasma glucose.


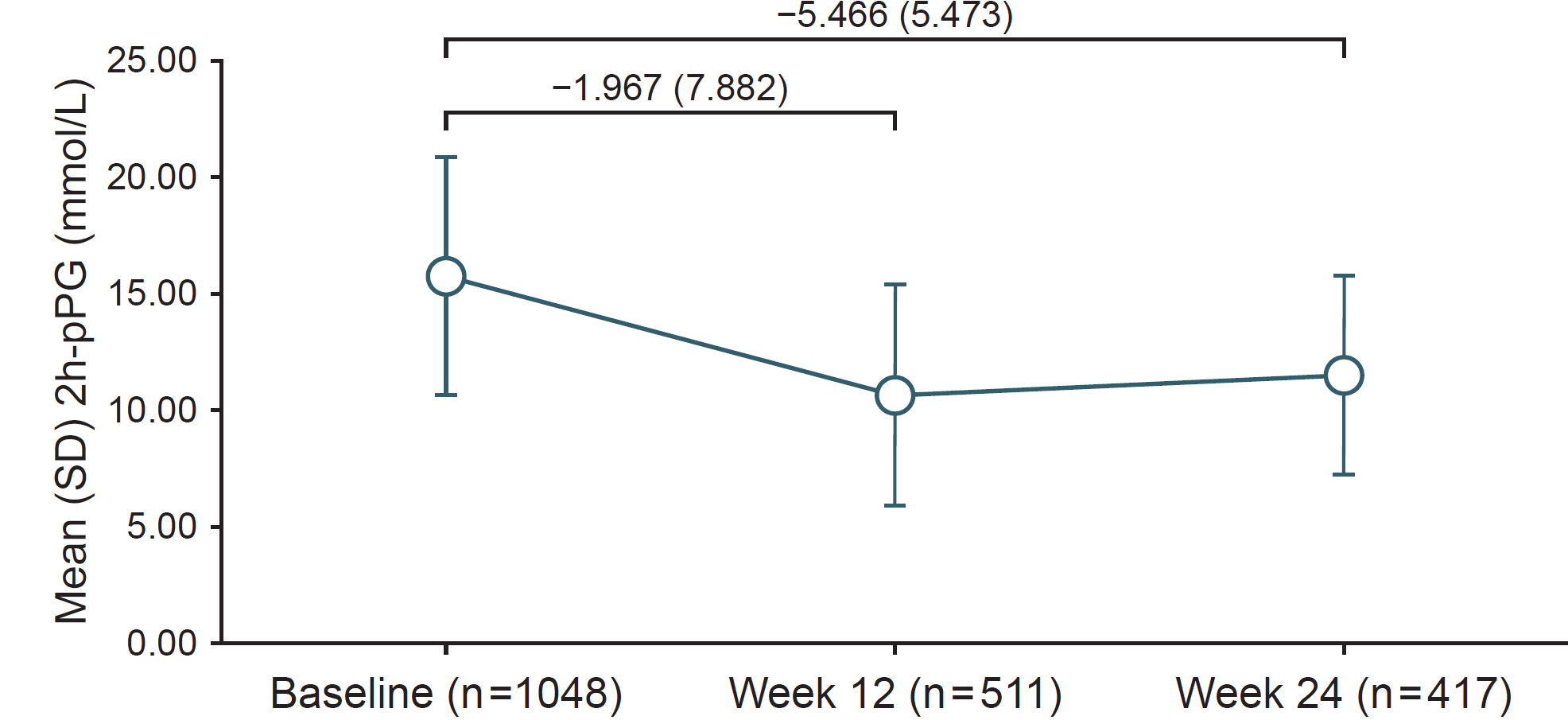


## Figure S4. Mean change from baseline in 2h-PPG (mmol/L) during the 24-week study follow-up (metabolic analysis set).

Error bars represent SD. 2h-PPG, 2-hour postprandial plasma glucose

**
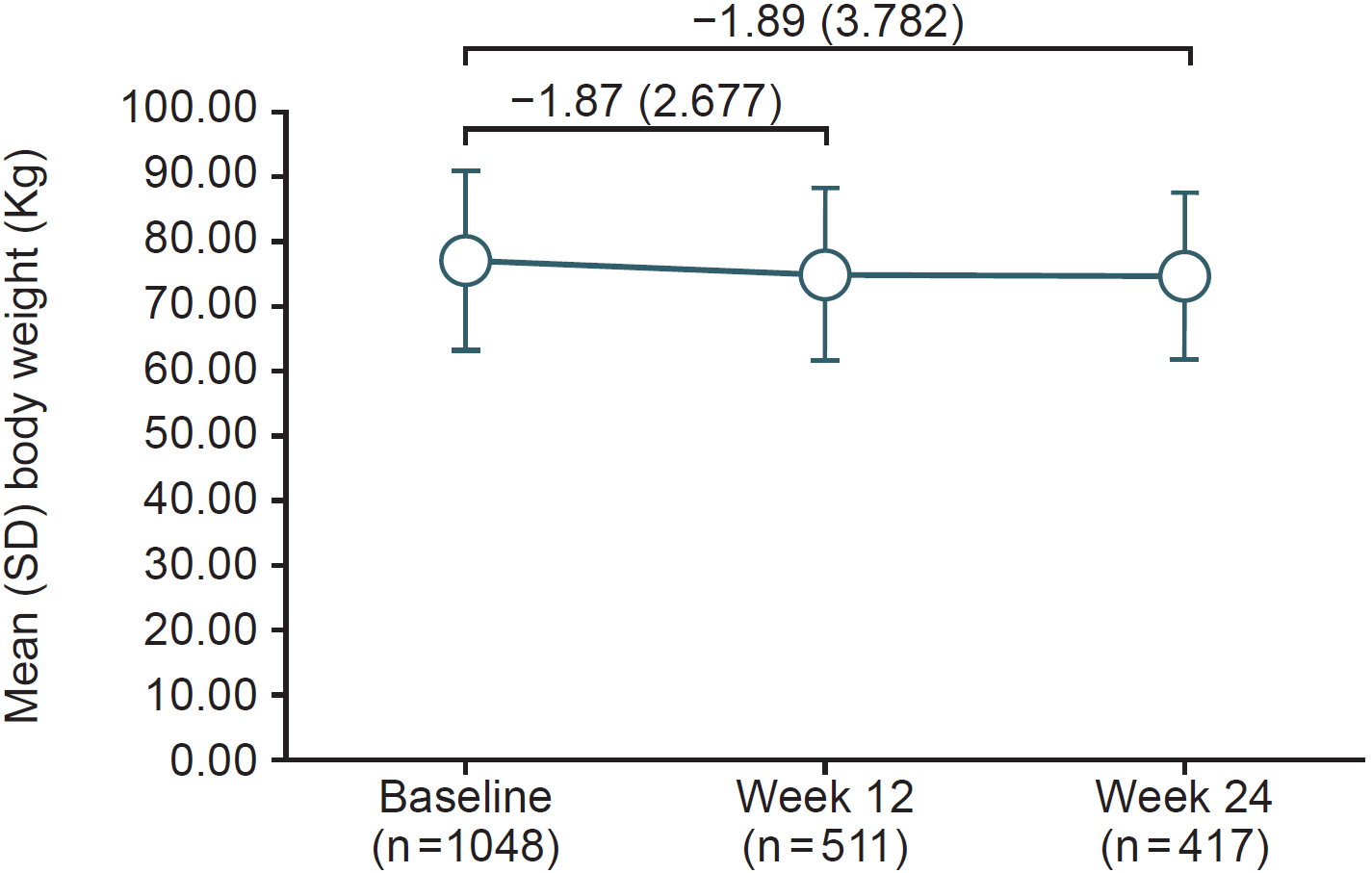
**

## Figure S5. Mean change from baseline in body weight (Kg) during the 24-week study follow-up (metabolic analysis set).

Error bars represent SD.

**
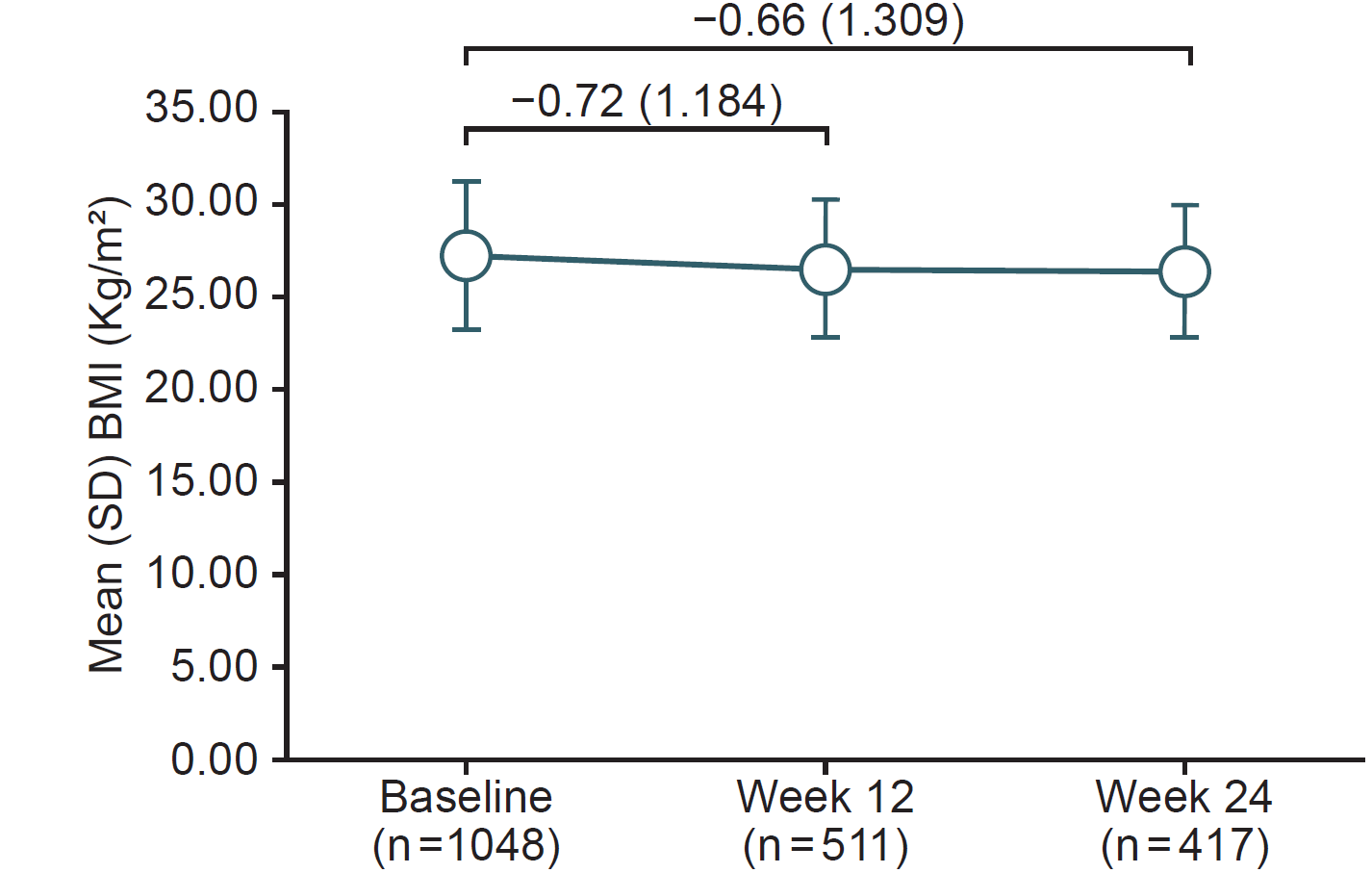
**

## Figure S6. Mean change from baseline in BMI (Kg/m^2^) during the 24-week study follow-up (metabolic analysis set).

Error bars represent SD. BMI, body mass index.

**
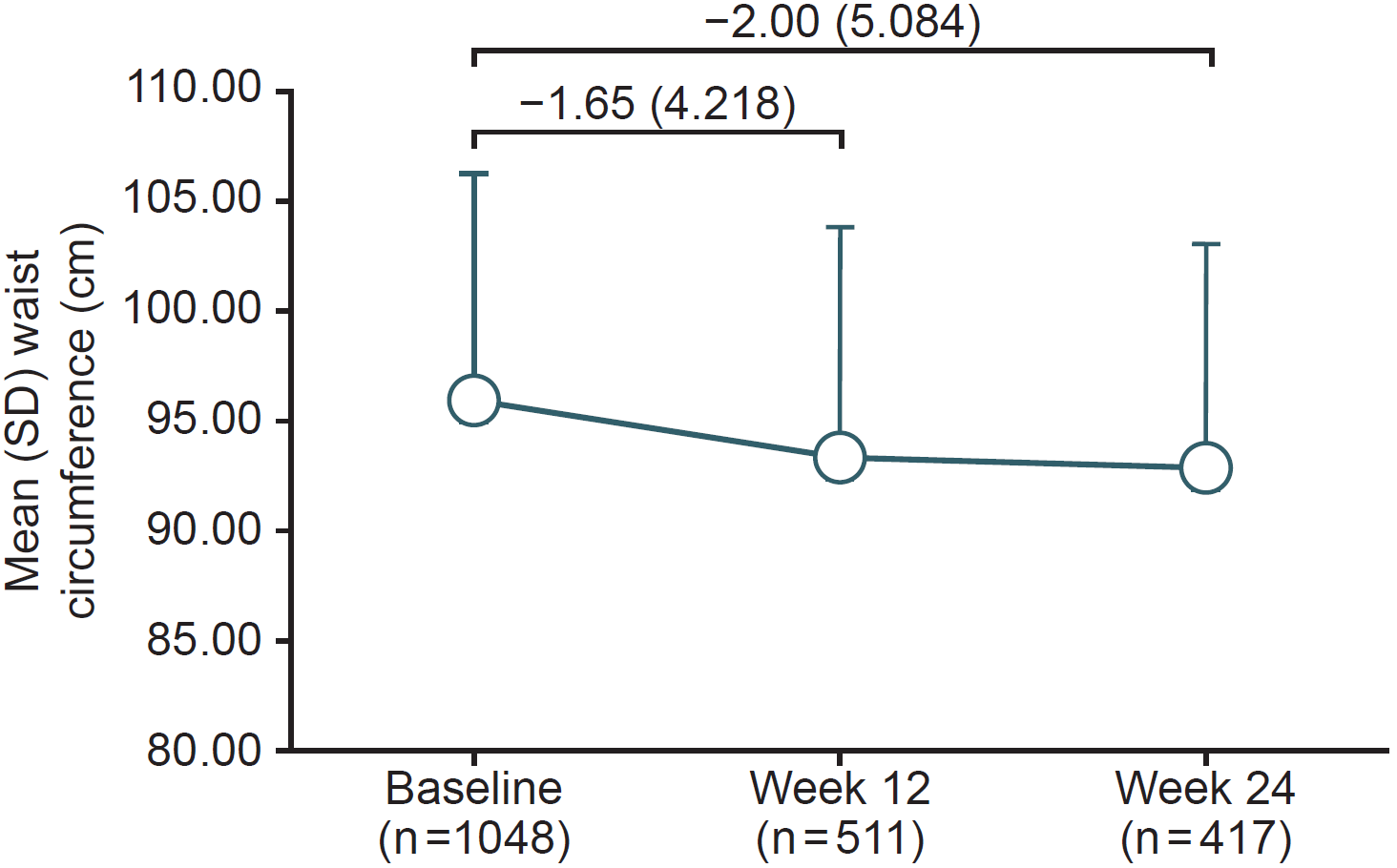
**

## Figure S7. Mean change from baseline in waist circumference (cm) during the 24-week study follow-up (metabolic analysis set).

Error bars represent SD.

**
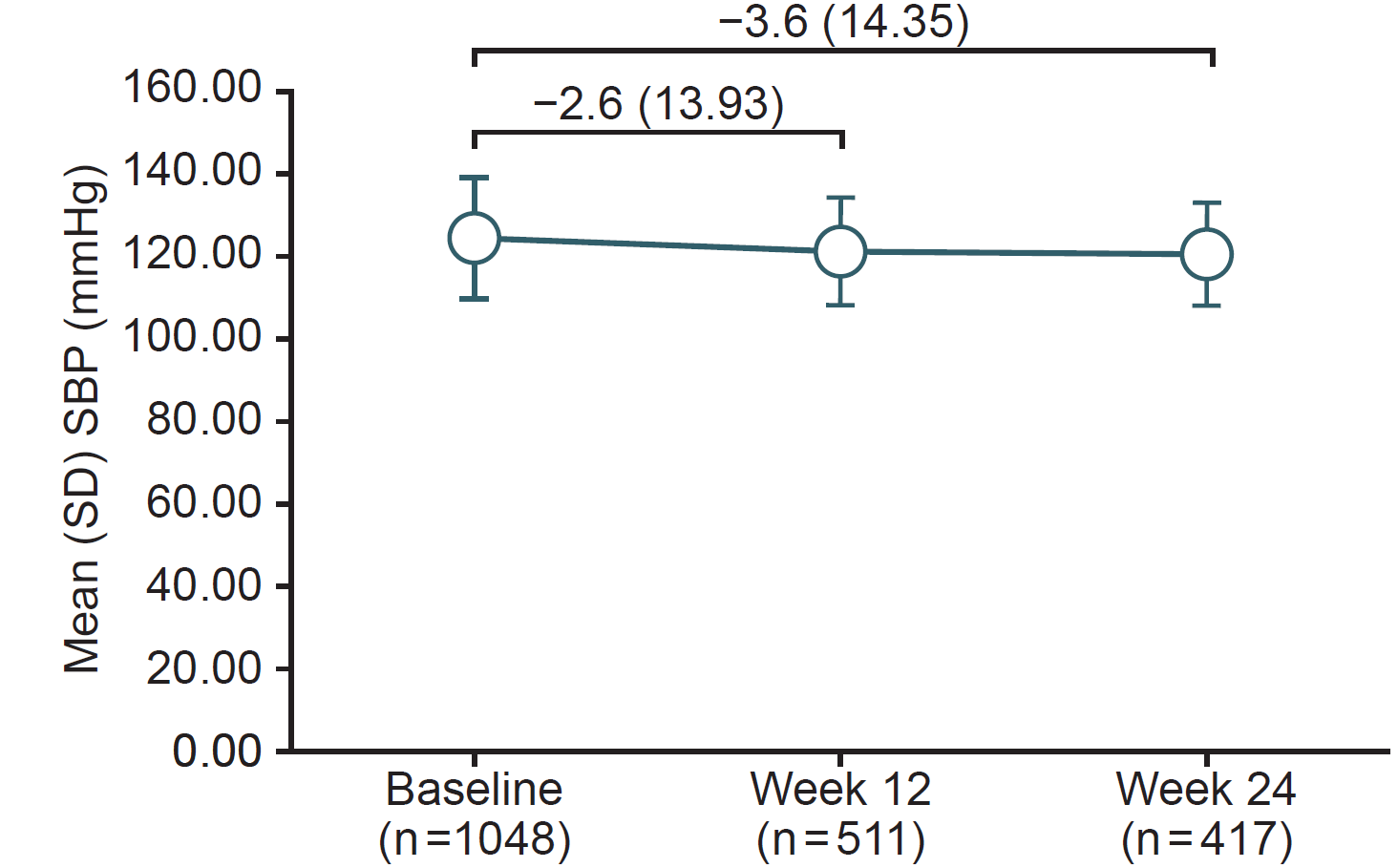
**

## Figure S8. Mean change from baseline in SBP (mmHg) during the 24-week study follow-up (metabolic analysis set).

Error bars represent SD. SBP, systolic blood pressure.

**
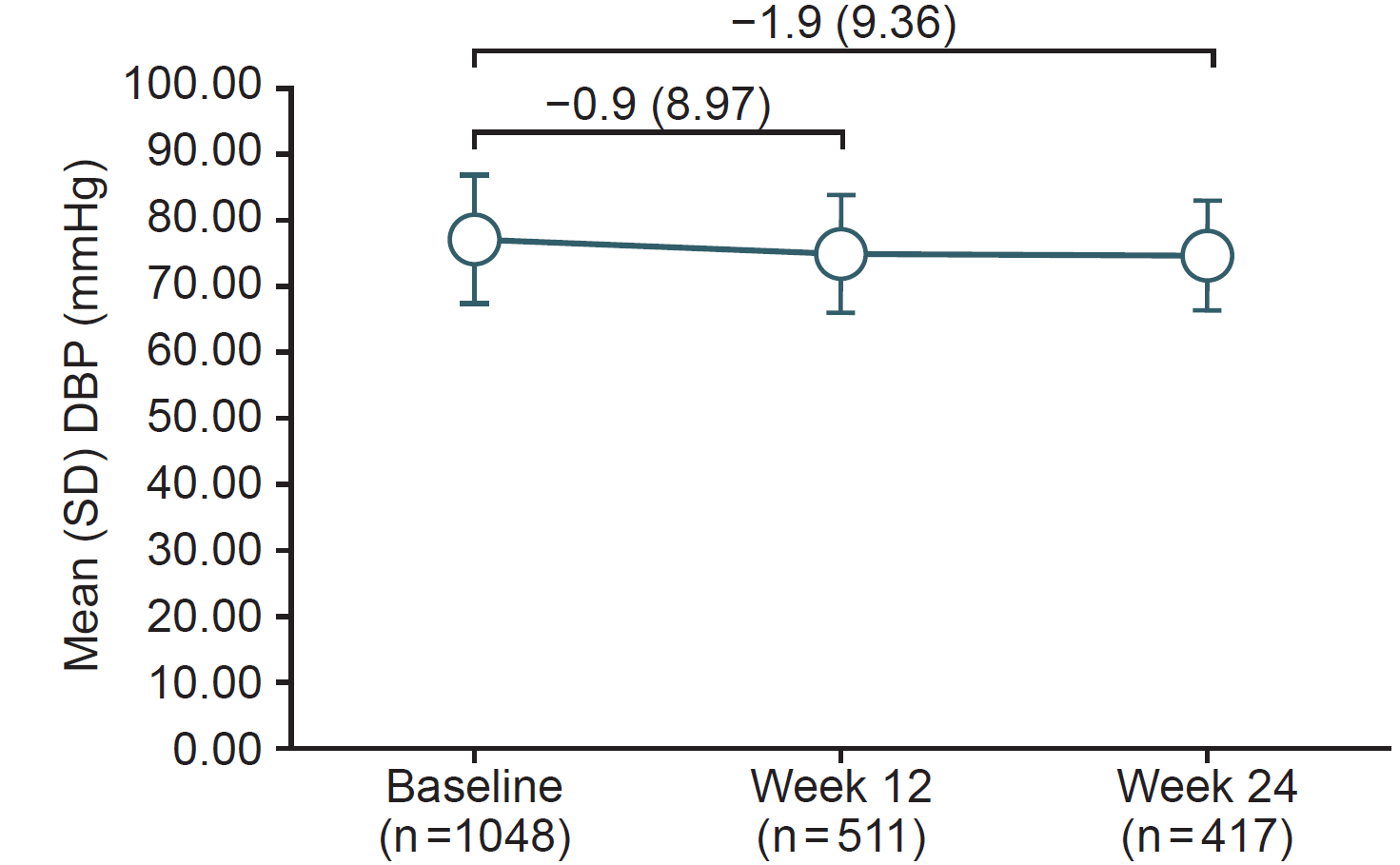
**

## Figure S9. Mean change from baseline in DBP (mmHg) during the 24-week study follow-up (metabolic analysis set).

Error bars represent SD. DBP, diastolic blood pressure

**
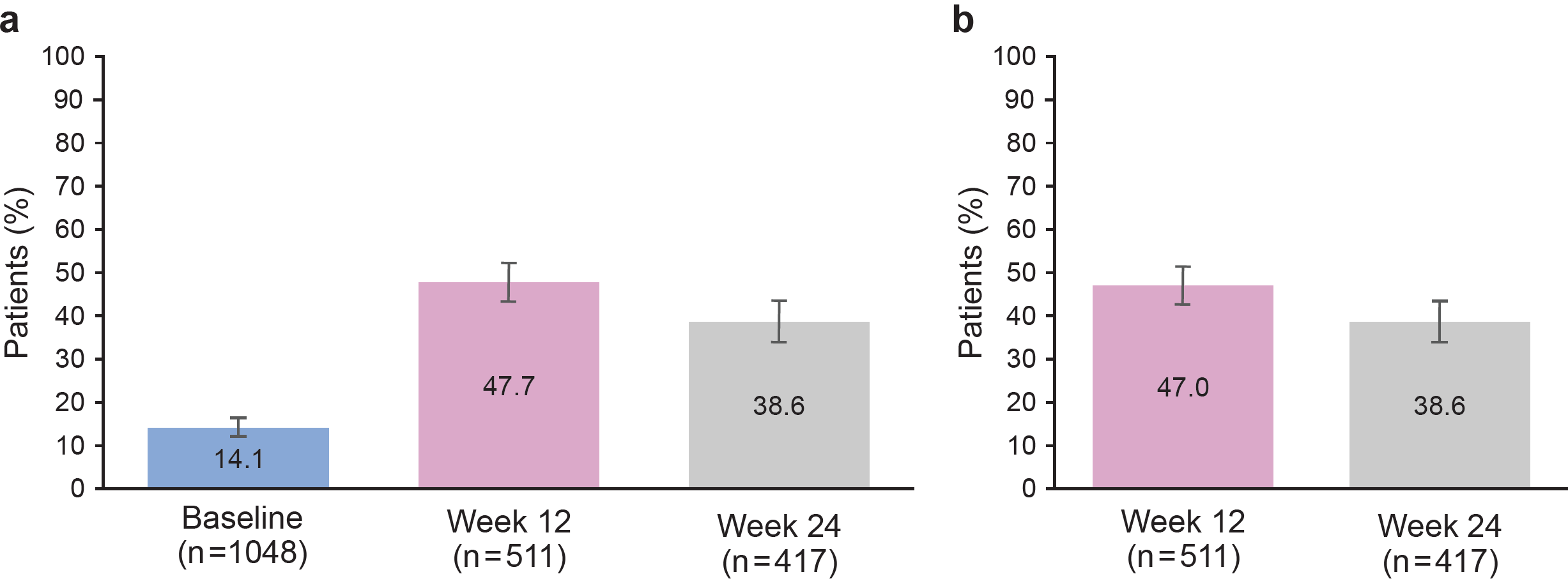
**

## Figure S10. Proportion of patients achieving HbA1c <7.0% throughout the 24-week study follow-up (metabolic analysis set).

(a) Proportion of patients achieving HbA1c <7.0%; (b) Proportion of patients achieving HbA1c <7.0% and without hypoglycaemia.

Error bars represent 95% CIs.
